# Avian Influenza in Wild Birds, Central Coast of Peru

**DOI:** 10.3201/eid1506.080981

**Published:** 2009-06

**Authors:** Bruno M. Ghersi, David L. Blazes, Eliana Icochea, Rosa I. Gonzalez, Tadeusz Kochel, Yeny Tinoco, Merly M. Sovero, Stephen Lindstrom, Bo Shu, Alexander Klimov, Armando E. Gonzalez, Joel M. Montgomery

**Affiliations:** Universidad Nacional Mayor de San Marcos, Lima, Peru (B.M. Ghersi, E. Icochea, R.I. Gonzalez, Y. Tinoco, A.E. Gonzalez); United States Naval Medical Research Center Detachment, Lima (B.M. Ghersi, D.L. Blazes, T. Kochel, Y. Tinoco, M.M. Sovero, J.M. Montgomery); Centers for Disease Control and Prevention, Atlanta, Georgia, USA (S. Lindstrom, B. Shu, A. Klimov, J.M. Montgomery)

**Keywords:** Avian influenza, influenza, wild birds, Peru, South America, animal surveillance, vector-borne infections, dispatch

## Abstract

To determine genotypes of avian influenza virus circulating among wild birds in South America, we collected and tested environmental fecal samples from birds along the coast of Peru, June 2006–December 2007. The 9 isolates recovered represented 4 low-pathogenicity avian influenza strains: subtypes H3N8, H4N5, H10N9, and H13N2.

Limited data are available on genotypes of avian influenza virus (AIV) circulating among wild birds in South America (*1*–*6*). To determine whether AIVs are present and circulating in wild birds in South America, we collected and examined bird environmental fecal samples.

## The Study

From June 2006 through December 2007, environmental fecal samples were collected from resident and migratory wild birds from 4 wetland areas along the central coast of Peru (Figure) and tested for evidence of avian influenza. Each wetland was visited 1×/week for 6 weeks; after a sampling period was completed, we would sample another wetland. Each wetland was sampled at least 2 times, with the exception of Villa, which was sampled only 1 time.

**Figure:**
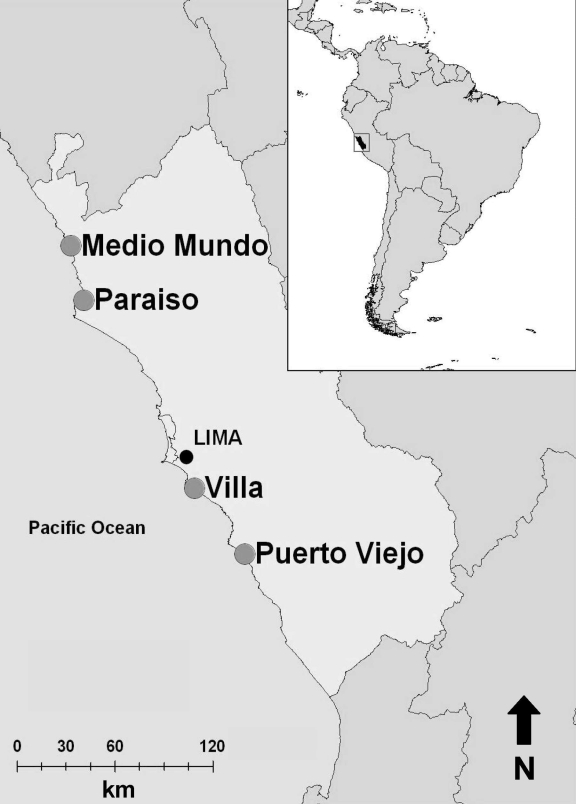
Locations of 4 sites (large circles) along coast of Peru where wild bird fecal samples were collected, June 2006–December 2007.

Bird colonies were identified from 7 am to noon and observed for 15–20 min by persons experienced in bird identification. Size and accessibility to the bird colonies affected which species were sampled. We focused on collecting samples from single-species flocks; however, mixed flocks were also sampled and identified to the family level or as a mix of species. Immediately after the colony left the resting area, fresh fecal samples were collected. Each sample was collected with a sterile-tipped applicator, placed in a cryovial containing transport media (RPMI 1640 with 5% bovine serum albumin; Sigma-Aldrich, St. Louis, MO, USA) in a Styrofoam box with ice packs at ≈4°C, and transported to the laboratory to be processed within 24 h of collection.

Samples were processed in the Avian Pathology Laboratory at the Veterinary School, San Marcos University. Virus isolation was conducted by standard methods (*7*). Samples were pooled according to species, date, and wetland; each pool consisted of 8–12 samples. Pools were centrifuged and filtered before inoculation into the allantoic cavity of 5 specific-pathogen-free 9-day-old chicken embryos. Eggs were incubated for 6 d; survival was checked daily. Allantoic fluid of each egg was tested for hemagglutinating agents by a direct hemagglutination assay (*7*). First-passage negative pools were passaged a second time to confirm absence of avian influenza. All hemagglutination-positive allantoic fluids were tested by antigen capture tests for influenza virus A and sent to the US Naval Medical Research Center Detachment, Peru, for further molecular characterization. Allantoic fluids were also sent to the US Centers for Disease Control and Prevention (Atlanta, GA, USA) for virus typing and sequencing. RNA extracts were prepared from 100 μL of allantoic fluids with the MagNA Pure Compact automated RNA extraction system (Roche Applied Science, Indianapolis, IN, USA). One-Step RT-PCR (Invitrogen, Carlsbad, CA, USA) was used to amplify hemagglutinin (HA) and neuraminidase (NA) genes with universal HA and NA oligonucleotide primers (*8*). Amplicons were purified by agarose gel electrophoresis followed by purification using the MiniElute Gel Extraction kit (QIAGEN, Valencia, CA, USA), then sequenced on an automated Applied Biosystems 3730 system (Foster City, CA, USA) using cycle sequencing dye terminator chemistry. Each gene segment sequence was analyzed by BLAST (www.ncbi.nlm.nih.gov/blast/Blast.cgi) analysis against the Influenza Sequence Database of Los Alamos National Laboratories (Los Alamos, NM, USA) for identification of avian influenza genotype.

A total of 2,405 samples, representing 27 species, were processed and analyzed (Table 1). Nine AIV isolates, from 7 species, representing 5 avian families, were recovered from 3 of the 4 wetlands sampled. Hemagglutinin subtypes H3, H4, H10, and H13 and neuraminidase subtypes N2, N5, N8, and N9 were identified (Table 2).

**Table 1:** Bird species sampled along central coast of Peru, June 2006–December 2007

Family	Species	Wetland, no. sampled				Total*
		Medio Mundo	Puerto Viejo	Paraiso	Villa	
Anatidae	*Anas bahamensis*		44		10	54
	*Anas cyanoptera*		37		20	57
	*Oxyura ferruginea*	10				10
	Mix†	46	38	115	30	229
Ardeidae	*Ardea alba*	10	14			24
	*Egretta thula*	8	37			45
	*Egretta caerulea*		10			10
	*Nycticorax nycticorax*		4			4
	Mix†	43	10	134	10	197
Laridae	*Larus pipixcan*	38	24	20	10	92
	*Larus modestus*	67	24		10	101
	*Larus dominicanus*	10				10
	*Larus belcheri*				20	20
	Mix†	53	16	65		134
Rinchopidae	*Rynchops niger*	79	19	10		108
Scolopacidae	*Calidris mauri*			10	21	31
	*Calidris alba*	105	23	41	50	219
	*Calidris pusilla*	31				31
	*Arenaria interpres*		61		20	81
	*Numenius phaeopus*		35	10	40	85
Charadridae	*Charadrius vociferus*		6	10		16
	*Charadrius semipalmatus*			10	10	20
	*Pluvialis squatarola*	9				9
Recurvirostridae	*Himantopus mexicanus*		3		10	13
Threskiornithidae	*Plegadis ridgwayi*			39		39
Rallidae	*Gallinula chloropus*		30			30
	*Fulica ardesiaca*	21	39	10		70
Pelecanidae	*Pelecanus occidentalis thagus*	86		56		142
Phalacrocoracidae	*Phalacrocorax brasilianus*	174	99	70	39	382
Haematopodidae	*Haematopus paliatus*	108	34			142
Total		898	607	600	300	2,405

*Does not represent the exact number because some were included in the mixed samples. †>2 species from same family.

**Table 2:** Birds with positive avian influenza virus test results, central coast of Peru, June 2006–December 2007

Date	Wetland	Common name (taxonomic name)	Hemagglutinin	Neuraminidase
2006 Oct 17	Puerto Viejo	Ruddy turnstone (*Arenaria interpres*)	H10	N9
2006 Oct 24	Puerto Viejo	Ruddy turnstone (*Arenaria interpres*)	H10	N9
2006 Nov 6	Puerto Viejo	White-cheeked pintail (*Anas bahamensis)* and cinnamon teal (*Anas cyanoptera*)	H3	N8
2006 Nov 7	Puerto Viejo	American oystercatcher (*Haematopus palliates*)	H10	N9
2007 Feb 6	Medio Mundo	White-cheeked pintail (*Anas bahamensis)* and cinnamon teal (*Anas cyanoptera)*	H4	N5
2007 Feb 13	Medio Mundo	White-cheeked pintail (*Anas bahamensis)* and cinnamon teal (*Anas cyanoptera)*	H4	N5
2007 Feb 13	Medio Mundo	Peruvian pelican (*Pelecanus occidentalis thagus*)	H4	N5
2007 Nov 20	Paraiso	Whimbrel (*Numenius phaeopus*)	H13	N2
2007 Nov 20	Paraiso	Dominican gulls (*Larus dominicanus*)	H13	N2

For the samples from Medio Mundo, sequences of the HA gene were 100% identical; however, the NA genes differed by 1 nt. Similarly, for the samples from Paraiso, the HA genes from both bird species were 100% identical, but the NA genes differed by 4 nt. From the Puerto Viejo samples, 2 different influenza virus A subtypes were recovered: H3N8 in ducks and H10N9 in 2 ruddy turnstones and an American oystercatcher. The HA and NA genes of the H10N9 were 100% identical in 3 isolates from 2 species. All samples from Villa were negative.

## Conclusions

We isolated no highly pathogenic AIVs; however, several low-pathogenicity avian influenza (LPAI) strains were identified. AIVs were recovered from both migratory and resident (nonmigratory) birds. Each strain was first isolated from a migratory bird and, in most instances, at least 1 week thereafter from resident species. Although this finding seems to support unidirectional transmission of the virus from migratory to resident birds, further research should be conducted to test this hypothesis. Furthermore, although subtype H13 is considered restricted to gulls (*9*), we isolated this subtype from a flock purely of whimbrels; this finding may have resulted from a spillover event, or perhaps gulls are not the sole reservoir species for this strain. Orders Charadriiforme and Anseriforme have a greater isolation index than other orders (*9*), and subtype patterns observed in this study appear to be the same as those observed in North America.

Migrating birds make frequent stopovers when moving between breeding and nonbreeding areas (*10*). The strains identified in our study may have been carried from Alaska and Canada through the continental United States, perhaps disseminating these viruses on their stopover sites before arriving in Peru. Thus, in the future, these strains may arise from within other countries along the north-to-south flyway.

We identified positive samples at the beginning and end of the migratory season, as has been found in previous studies (*9*–*11*). Our inability to detect viruses throughout the year, over multiple seasons, may be partially explained by insufficient sample size. The sample size was based on the assumption that the prevalence rate of circulating virus should be at least 1%; however, the rate may be lower.

The proximity of the Peru wetlands to human habitation, swine farms, and chicken farms could represent a risk for transmission of influenza viruses from wild birds to poultry, humans, and pigs. Wild birds were suspected to be the source of the avian influenza outbreak in Chile in 2002. This hypothesis was later supported by Spackman et al. (*6*), who identified an LPAI virus from a cinnamon teal; this virus was almost an exact match to the LPAI strain circulating among poultry during the Chile outbreak. Although the strains identified in our study were LPAI, a genetic shift, which likely occurred during the Chile outbreak, is possible.

Another study limitation is that field identification of bird species is sometimes difficult and can result in misidentification. Although most birds in a flock are generally of the same species, it is not uncommon to find additional species, albeit few, intermingled within the flock (e.g., calidrids and gulls). Therefore, bird sources of the fecal samples collected may have been misidentified. Although the probability that a sample may be recovered from 1 rogue species within a group of a different species is low, to minimize this uncertainty we assigned a few samples to the family level or as a mixture of 2–3 species. We were also unable to determine the prevalence of avian influenza in the wetlands sampled because environmental sampling limited our ability to determine whether multiple samples were from the same bird.

Our systematic evaluation of avian influenza strains among migratory and resident aquatic bird populations in South America used a cost-effective and efficient surveillance method to monitor AIV in bird populations at a specific location (*12*). Although all isolates were LPAI strains, our data support the hypothesis that migratory birds can serve as vectors for the spread of AIV among nonmigratory species. More data are needed to determine the role that migratory birds can play in spreading AIVs throughout the region.
